# Bone metabolism – an underappreciated player

**DOI:** 10.1038/s44324-024-00010-9

**Published:** 2024-07-01

**Authors:** In Ah Choi, Akio Umemoto, Masataka Mizuno, Kyung-Hyun Park-Min

**Affiliations:** 1 Arthritis and Tissue Degeneration Program, David Z. Rosensweig Genomics Research Center, Hospital for Special Surgery, New York, NY 10021 USA; 2 Department of Internal Medicine, College of Medicine, Chungbuk National University, Cheongju, Chungcheongbuk-do 28644 Republic of Korea; 3 Department of Medicine, Weill Cornell Medical College, New York, NY 10065 USA; 4 BCMB allied Program, Weill Cornell Graduate School of Medical Sciences, New York, NY 10021 USA

**Keywords:** Cell biology, Endocrinology

## Abstract

Bone is constantly being remodeled, and this process is orchestrated by a dynamic crosstalk of bone cells, including osteoclasts, osteoblasts, and osteocytes. Recent evidence suggests that cellular metabolism plays a crucial role in the differentiation and function of bone cells and facilitates the adaptation of bone cells to changes in the bone microenvironment. Moreover, bone affects whole-body energy metabolism. However, it is not yet completely understood how different cells in bone coordinate metabolic processes under physiological conditions, and how altered metabolic processes in bone cells contribute to pathological conditions where the balance among bone cells is disrupted. Therefore, gaining a better understanding of the distinct metabolic requirements of bone cells can provide crucial insights into the dysfunction of bone cells in pathological conditions and can be used to identify new therapeutic approaches to treat bone diseases. Here, we discuss recent advances in understanding metabolic reprogramming in bone cells.

## Introduction

Bone is a vital organ that is constantly remodeled throughout the lifetime^1^. Bone consists of cortical bone that surrounds a central bone marrow cavity and trabecular bone that traverses a bone marrow cavity. Bone includes various components, including bone cells such as osteoclasts, osteoblasts, and osteocytes, along with the extracellular matrix. Bone matrix is composed of organic and inorganic components. The breakdown of bone by osteoclasts releases ions, organic acids, and proteases and provides metabolic substrates to fuel metabolic anabolism in bone resident cells, including stem cells, immune cells, and bone cells. Liberated phosphate, magnesium, and calcium play a critical role in cellular metabolism by providing co-factors for metabolic enzymes and modifying metabolites.

Bone plays a central role in providing structural support to the body, protecting organs, and facilitating locomotion and sensory perception^1^. On the other hand, bone also serves additional functions^2^. Bone acts as storage for vital minerals and other essential ions^2,3^. Additionally, bone is a source of stem cells and bone marrow provides an ideal environment for the process of hematopoiesis. Hematopoiesis takes place at “niches” situated inside the bone marrow, within the central cavity of bones^4^. Importantly, bone is also considered to be an endocrine organ as bone releases various proteins, hormones, and growth factors into the circulation and affects other cells’ function and energy homeostasis^5,6^. However, these diverse functions of bone have been overlooked.

The bone microenvironment is constantly changing due to multiple factors such as aging, lifestyle, health conditions, nutritional status, and pathological conditions. Bone cells constantly undergo metabolic adaptation to counter these changes. Bone is affected by the metabolic status of individuals. However, the mechanisms behind this process and the bioenergetic characteristics of bone cells are not yet fully understood. It is crucial to comprehend the metabolic needs of various bone cells to gain a deeper understanding of bone diseases and create cell-specific treatments for bone cells. In this review, we aim to present an overview of the current knowledge on metabolic reprogramming in certain bone cells.

## Bone cells

**Fig. 1:**
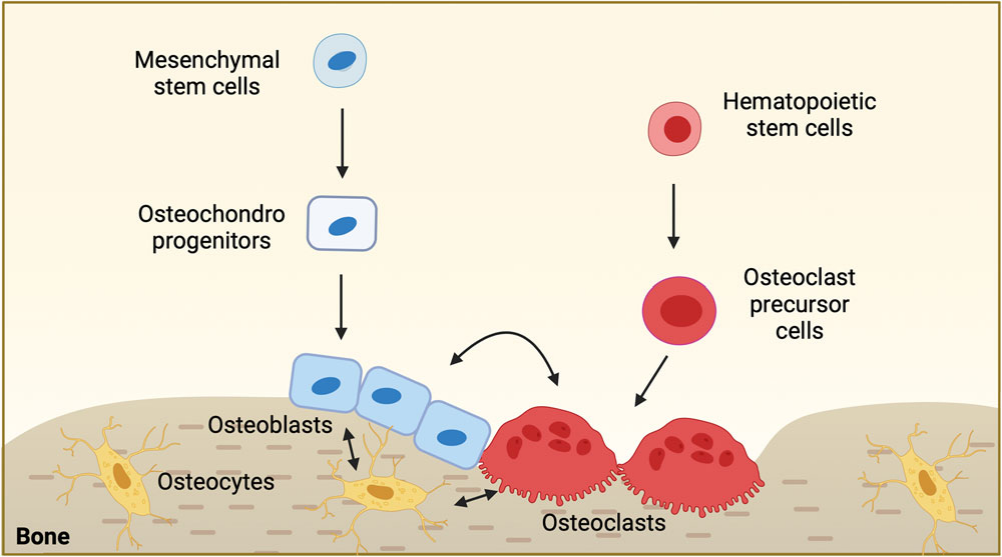
Bone remodeling. Bone remodeling is a fundamental process to maintain bone throughout life. It involves the removal of old bone tissue by osteoclasts and subsequent formation of new bone formation by osteoblasts. Maintaining a balance between these two types of cells is tightly regulated and essential for healthy bone homeostasis. Osteoclasts are multinucleated cells that originate from myeloid lineage cells. Osteoclasts are multinucleated cells that originate from hematopoietic stem cells. Osteoclast precursor cells fuse with each other to form these multinucleated cells. The formation of osteoclasts is activated by RANKL. On the other hand, mesenchymal stem cells differentiate into osteochodroprogenitors. Osteochondroprogenitor cells further differentiate into preosteoblasts that eventually mature into osteoblasts. A subpopulation of osteoblasts undergoes terminal differentiation to osteocytes which are embedded within the bone tissue. Osteocytes play a crucial role in controlling both osteoblasts and osteoclasts.

Bone is continuously being maintained through bone remodeling, a delicate process balanced between osteoclastic bone resorption and osteoblastic bone formation^7^. Osteoclasts, osteoblasts, and osteocytes are key cells involved in bone remodeling (Fig. 1). Old and/or damaged bone is constantly replaced with newly formed bone by the coordinated actions of bone cells. Bone remodeling plays a crucial role in maintaining and modifying bone structure, repairing micro-cracks and fractures, and maintaining blood calcium homeostasis. The process of bone remodeling is tightly regulated and is affected by numerous factors, such as metabolic hormones and the availability of nutrients. A disruption in this balance can lead to poor bone health and diseases.

### Osteoclasts

Osteoclasts are multinucleated cells responsible for bone resorption and are derived from precursor cells in a myeloid cell origin^8–10^. Osteoclasts were traditionally considered terminally differentiated cells. However, recent studies have discovered that they are long-lived, constantly renewed by circulating precursors^11^, and recycled by fusion and fission of osteomorphs^12^. High energy is required for cell-cell fusion during osteoclast differentiation and bone resorption. Osteoclasts not only resorb bone but also play a role in regulating additional functions, including hematopoiesis, stem cell mobilization, and specific immune functions^13^. Dysregulated osteoclast formation and activity are linked to various bone diseases such as osteoporosis, osteopetrosis, rheumatoid arthritis, bone metastasis, and periodontitis. M-CSF (macrophage colony-stimulating factor) and RANKL (receptor activator of nuclear factor kB ligand) are key factors for osteoclast differentiation and function. M-CSF is an essential growth factor for the differentiation and survival of myeloid cells^14^. RANKL is a key driver of osteoclast differentiation and has been used as a therapeutic target for bone loss. Denosumab, an anti-human RANKL monoclonal antibody, is an FDA-approved anti-resorptive drug for osteoporosis and pathological bone loss by competitively inhibiting the RANKL-RANK interaction^15,16^. However, in certain pathological conditions, such as inflammatory arthritis, RANKL-independent osteoclastogenesis has been observed (reviewed in ref. ^17^).

### Osteoblasts

Osteoblasts promote mineralization and bone formation by synthesizing osteoid and secreting bone matrix. The skeletal lineage cells are involved in osteogenesis and undergo three distinct differentiation stages: osteoprogenitor, preosteoblasts, and osteoblasts^18^. During bone remodeling, osteoblasts require ATP to increase collagen biosynthesis and mineralization.

Mesenchymal stem cells (MSCs) can differentiate into multiple cells in the skeletal system, including osteoblasts, osteocytes, chondrocytes, fibroblasts, adipocytes, stromal cells, and myoblasts^19^. Osteoblasts can be derived from multicomponent MSCs and skeletal stem cells (SSCs)^20^. Various stem cell-specific markers have been identified, and recent studies suggest that osteoblasts in different bones may have originated from site-specific stem cells. Osteochondroprogenitor cells originate from precursors that express SOX9 and can differentiate into chondrocyte and osteoblast-lineage cells^21^. After the synthesis of type I collagen, the main component of bone matrix, the Runt-related transcription factor 2 (Runx2) is expressed in preosteoblasts. Runx2 is a key determinant of osteoblast differentiation^22^. Runx2 is required for the commitment of preosteoblasts to osteogenic lineage and induces the expression of major bone matrix genes, leading to the formation of immature bone^22^. During the maturation of preosteoblasts, osterix (OSX, also known as Sp7), which is induced by the WNT-β catenine signaling, directs the differentiation of preosteoblasts to osteoblasts^23^. Mature osteoblasts differentiate into lining cells or osteocytes or undergo apoptosis.

### Osteocytes

Osteocytes, which are derived from a subset of osteoblasts, are terminally differentiated cells embedded within the mineralized bone matrix^24,25^. Osteocytes are the most abundant cells in bone and are a master regulator of bone remodeling. Osteocytes respond to hormonal and mechanical signals and orchestrate bone homeostasis by controlling osteoblasts and osteoclasts. Mature osteocytes selectively secrete molecules such as Dickkopf-related protein 1 (DKK1), osteoprotegerin (OPG), and sclerostin. Osteocytes are a mechanosensory cell, and the mechanical loading regulates the lifespan and the gene expression of osteocytes^26^. There are several mechanosensors in osteocytes, including the osteocyte cytoskeleton, dendritic processes, integrin-based focal adhesions, connexin/pannexin channels, primary cilium, ion channels, and extracellular matrix^27^. During the mechanotransduction process, osteocytes decrease the expression of sclerostin, leading to increased bone formation through the activation of WNT signaling in osteoblasts.

## Bone cells and energy metabolism

**Fig. 2:**
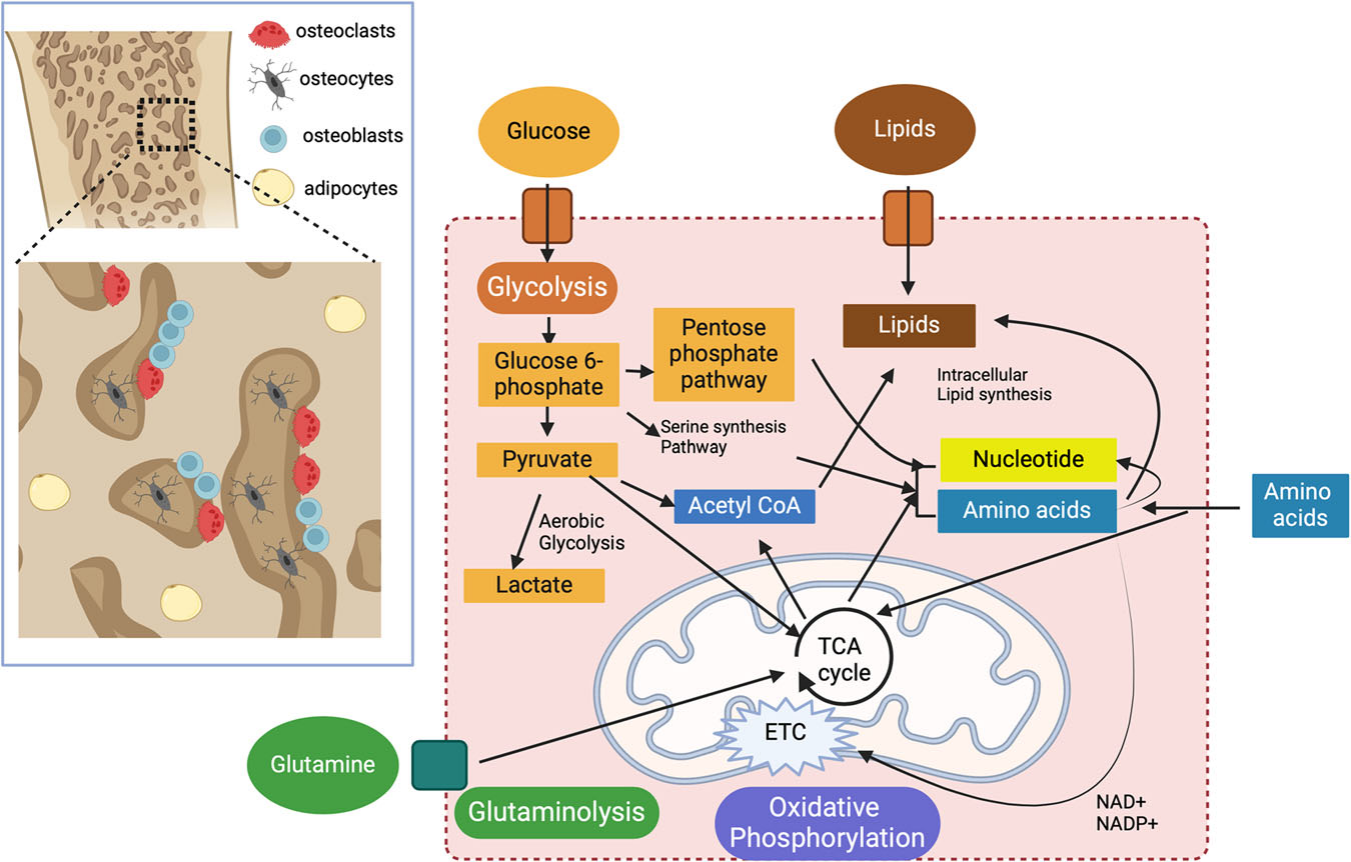
Energy metabolism. In bone cells, energy is generated by interconnected metabolic pathways, including glycolysis, the tricarboxylic acid cycle (TCA cycle), glutaminolysis, and oxidative phosphorylation (OXPHOS). Glucose is the primary energy and carbon source for bone growth and development. The transportation of glucose by glucose transporter (GLUT) proteins occurs spontaneously along a concentration gradient, without the need for energy. Once inside the cell, glucose is converted to glucose-6-phosphate (G6P) by hexokinase, which can then be used to generate glycogen or enter the glycolytic pathway to generate pyruvate. Glycolysis coupled with lactic acid fermentation and oxidative phosphorylation in the mitochondria produces adenosine triphosphate (ATP), the most important high-energy chemical in the body. Lactate dehydrogenase (LDH) can also convert glucose to lactate independently of oxygen through aerobic glycolysis. The glucose 6-phosphate, produced early in glycolysis, can be directed towards the pentose phosphate pathway, leading to nucleotide synthesis, or it can contribute to the serine synthesis pathway, which is important for amino acid production. The serine biosynthetic pathway, which generates methyl groups for DNA and histone methylation, is also vital for one-carbon metabolism. OXPHOS occurs in the mitochondria and is the final metabolic pathway for all oxidative steps in carbohydrates, amino acids, and fatty acid catabolism. The electron carriers NAD+ and NADP+ drive the ETC for oxidative phosphorylation, resulting in the production of ATP. The Krebs cycle, also known as the tricarboxylic acid cycle (TCA), is the primary metabolic pathway that produces energy in cells and provides reduced co-factors and metabolic intermediates. Acetyl CoA, derived from pyruvate, is also supplied by the TCA cycle and acts as a central hub to promote intracellular lipid synthesis. Parallel to these processes, glutamine is metabolized through glutaminolysis, contributing to the pool of substrates necessary for energy production and biosynthesis in bone cells. TCA cycle tricarboxylic acid cycle, Acetyl CoA acetyl coenzyme A, NAD+ nicotinamide adenine dinucleotide, NADP+ nicotinamide adenine dinucleotide phosphate, ETC electron transport chain, ATP adenosine triphosphate.

Bone cells use various metabolic pathways, such as carbohydrate metabolism, energy metabolism, lipid metabolism, nucleotide metabolism, and amino acid metabolism, to obtain energy for their survival, differentiation, and function (Fig. 2).

### Glycolysis

#### Osteoclasts and glycolysis

Osteoclast formation requires both glycolytic and oxidative metabolism to meet high-energy requirements. Glycolysis is crucial for osteoclast activity, especially when resorbing the bone matrix^28–31^. During osteoclastogenesis, RANKL stimulation induces the expression of glycolytic genes and glucose uptake. Mature osteoclasts consume glucose as their primary source of nutrients^17^. Immunostaining experiments reveal that several key glycolytic enzymes are located in the actin ring of polarized mature osteoclasts that adhere to the bone surface^32^. Osteoclast precursor cells express glucose transporters, including GLUT1 and GLUT3, while GLUT1 is upregulated in mature osteoclasts^29^. GLUT1 deficiency in LysM Cre suppresses in vitro osteoclastogenesis while exhibiting an osteopetrotic bone phenotype only in female mice^33^, supporting the independent role of aerobic glycolysis in osteoclast differentiation. However, the mechanism for female-biased phenotype is not clear. Increased glycolysis leads to the accumulation of glycolytic intermediates that regulate osteoclastogenesis. High concentrations of fructose 1,6-bisphosphate, one of glycolytic intermediates, have been shown to inhibit RANKL-induced osteoclastogenesis and TRAP activity by inhibiting the NF-kB/NFATc1 pathway in mouse bone marrow (BM)-derived osteoclasts in vitro^34^. During osteoclastogenesis, glycolysis-derived lactate progressively increases and supports bone resorption^28^. The expression of lactate dehydrogenase (LDH), which catalyzes the conversion of pyruvate to lactate, is elevated in mature osteoclasts. Deletion of LDHA or LDHB subunits suppresses both glycolysis and mitochondrial respiration, leading to impaired fusion of osteoclast precursor cells^35^. Inhibition of glycolysis with 2-deoxy-glucose (2DG) suppresses osteoclastogenesis and bone resorption, whereas the addition of lactate and pyruvate restores impaired osteoclastogenesis. Additionally, individual glycolytic intermediates can be further utilized in other metabolic pathways^36^. Coactivator-associated arginine methyltransferase 1 (CARM1) mediates arginine methylation of PPP1CA and reprograms glucose metabolism in osteoclasts and osteoblasts by switching from oxidative phosphorylation to aerobic glycolysis, resulting in enhanced osteogenic differentiation and impaired osteoclast differentiation^37^.

As oxygen tension is very low in bone^38^, bone tissue exhibits a hypoxic nature. While there is conflicting evidence on the impact of hypoxia on osteoclasts, most studies indicate that hypoxia increases both osteoclast formation and activity and leads to an increase in the glycolytic flux in osteoclasts^39^. Hypoxia-inducible factor (HIF)-1 is a key regulator of cellular response to hypoxia and is stabilized in hypoxic microenvironments. When osteoclasts are formed under hypoxia, they exhibit increased expression of HIF-1 and glycolytic enzymes. Blocking glycolytic enzymes reduces acid secretion from hypoxic osteoclasts, and chemical inhibition of phosphofructokinase E1 (PFK1) and LDHA significantly reduces hypoxia-induced osteoclast formation^40^. Osteoclasts under hypoxic conditions increase ATP production by consuming more oxygen and using HIF-1α-dependent glucose uptake^41^. However, the role of HIF-1 proteins in osteoclasts remains controversial^42^. While knockdown of HIF-1α inhibits bone resorption by mature osteoclasts, studies show that osteoclast differentiation is not affected by the deficiency of HIF-1α^29,43^. Another study shows that hypoxia suppresses the copper metabolic domain containing 1 (COMMD1), which then leads to an increased expression of E2F1, a transcription factor that primarily controls the cell cycle in non-proliferating human osteoclast precursor cells (OCPs) in response to RANKL^44^. E2F1 increases the expression of genes in glycolysis. A recent study shows that RANKL-induced LSD1 stabilizes the HIF-1α protein in OCPs, leading to increased glycolysis in conjunction with E2F1, which is also upregulated by LSD1^45^. These observations indicate that increased glucose metabolism fuels bone resorption, contributing to osteoclast-mediated pathologies.

#### Osteoblasts and glucose metabolism

Glucose is an essential nutrient for osteoblast differentiation and bone formation, as demonstrated in early studies showing the rapid consumption of glucose by bone explants and isolated osteoblasts^46–48^. Glucose provides energy and a carbon source for bone-building block molecules. Studies using radiolabeled glucose analogs confirm significant uptake of glucose by mouse bones^49,50^. It is estimated that approximately 40% of ATP in immature osteoblasts and nearly 80% of ATP in mature osteoblasts are produced through glycolysis^51–53^. Osteoblasts express GLUT1, GLUT3, and GLUT4, which show distinct expression patterns and functions for osteoblast proliferation and differentiation^49,54–56^. GLUT1 is essential for osteoblast differentiation, and genetic studies in mice demonstrate that it is required for the osteoanabolic activity of WNT7b^49,57^. In addition, GLUT1 helps regulate RUNX2 protein expression by maintaining ATP levels to limit AMPK activation and preventing the proteasomal degradation of RUNX2^45^. However, glucose flux is not able to initiate bone formation when RUNX2 is not present, suggesting the feed-forward requirement between RUNX2 and GLUT1 for increased glucose uptake and matrix production in osteoblasts. However, the impact of GLUT1-mediated glucose uptake on osteoblast differentiation varies by differentiation stages and may be transient. GLUT1 deletion with Prx1 Cre shows severe limb shortening and impaired chondrocyte proliferation and hypertrophy^52^. GLUT1 deficiency with osteocalcin (OCN) Cre exhibits diminished bone mass, while GLUT deficiency with Col1 Cre displays a high bone mass^49^. GLUT4 regulates insulin-stimulated glucose uptake and in vitro osteoblast differentiation. GLUT4 deficiency with OCN Cre exhibits an increase in peripheral fat in association with hyperinsulinemia, β-cell islet hypertrophy, and reduced insulin sensitivity but shows normal bone architecture^55^, suggesting the redundant and transient role of GLUT proteins in osteoblast differentiation.

Lactate is a major byproduct of glycolysis in osteoblasts regardless of the presence of oxygen. Lactate production increases in primary osteoblasts during differentiation^58,59^. The Warburg effect (aerobic glycolysis) is observed in in vitro cultures of osteoblasts. Metabolic tracing with labeled glucose confirms that lactate is the predominant product of glycolysis in the cortical bone of mouse long bone in vivo^60^. Lactate promotes osteoblast differentiation by several mechanisms, including stabilizing HIF-1, enhancing the effect of PTH and acetylation of p300, and limiting the generation of reactive oxygen species (ROS) and oxidative stress. Moreover, lactate serves as a major carbon source of histone lactylation that is involved in promoting osteoblast differentiation^61^. Glycolysis in osteoblast lineage cells is known to be directly stimulated by various bone anabolic signals. Parathyroid hormone (PTH) stimulates both bone formation and bone resorption. PTH stimulates glucose consumption and lactate production during bone explants^48,62,63^ and in osteoblasts^54,63–67^. In a study using MC3T3-E1 cells, PTH was found to stimulate aerobic glycolysis through the activation of insulin-like growth factor (IGF) signaling^67^. Conversely, reducing aerobic glycolysis with dichloroacetic acid (DCA) markedly suppressed PTH-mediated bone anabolism, supporting a functional link between glycolysis and bone formation^67^. Other signals, including the wingless/int-1 (Wnt) pathway, the myokine irisin, and nitric oxide (NO), also regulate glycolysis in osteoblasts^68,69^. MiR-34a targets the expression of glycolytic enzymes, thereby impeding osteoblast differentiation in human MSCs^70^. Therefore, the activation of aerobic glycolysis is a crucial mechanism underlying the osteoanabolic effects of PTH and is regulated by various signals that are important for bone formation.

### Bone cells and oxidative phosphorylation

#### Osteoclasts and oxidative phosphorylation

Oxidative phosphorylation (OXPHOS) is one of the preferred bioenergetic pathways for supporting osteoclast differentiation. Inhibiting OXPHOS suppresses osteoclastogenesis^71^. Osteoclast differentiation is accompanied by an increase in the number of mitochondria^72^, an increased expression of enzymes involved in metabolic pathways, increased metabolites^73^, and increased OXPHOS^71^. Peroxisome proliferator–activated receptor-gamma coactivator 1β (PGC1β), mitochondrial DNA, or mitochondrial transcription factor (Tfam) can stimulate the mitochondrial biogenesis during osteoclastogenesis^74,75^. There is controversy regarding the prerequisite of mitochondrial biogenesis for osteoclast differentiation, as there are conflicting results regarding the bone phenotype of myeloid-specific PGC1β-deficient mice^76,77^. OXPHOS generates ATP for osteoclast differentiation while RANKL-induced OXPHOS is diminished in mature osteoclasts^71^. The MYC-ERRa axis serves as an upstream regulator of OXPHOS without regulating mitochondrial biogenesis, while MYC also activates NFATc1 and the osteoclastogenic program^71^. The electron transport chain (ETC) complexes in mitochondria produce mitochondrial reactive oxygen species (ROS) and intermediate metabolites that regulate a variety of cellular functions. Inhibiting the ETC complexes or the mitochondrial network also suppresses osteoclastogenesis, supporting the significance of OXPHOS in osteoclast differentiation (reviewed in refs. ^17,78^). Several studies suggest that alternative metabolic pathways, such as fatty acid oxidation or amino acid metabolism, may be necessary for OXPHOS in osteoclasts. L-arginine suppresses TNFα-mediated osteoclast formation and protects against inflammatory bone loss by perturbing energy metabolism from glycolysis to oxidative phosphorylation^79^. Although lactate-producing glycolysis occurs independent of OXPHOS in osteoclasts^33^, knockdown of LDHA or LDHB suppresses osteoclastogenesis and mitochondrial respiration^35^, suggesting the significant but uncharacterized role of the crosstalk between glycolysis and OXPHOS in osteoclasts.

#### Osteoblasts and oxidative phosphorylation

Although glycolysis is the primary mode of energy metabolism in osteoblasts, there is an increase in OXPHOS along with the expansion of the mitochondrial network during osteoblast differentiation^58,80,81^. However, the use of OXPHOS-driven ATP generation in osteoblasts varies based on cell differentiation stages or cell sources. In bone marrow stromal cells (BMSCs), oxidative phosphorylation provides ATP at the early stage of osteoblast differentiation but glycolysis plays a significant role in energy production in mature osteoblasts^53,58^. A recent study analyzes cellular metabolism in MC3T3-e1 cells at the single-cell level using two-photon microscopy and shows that osteoblasts undergoing mineralization may use OXPHOS as an energy souce^82^. During osteogenic differentiation, human mesenchymal stem cells (MSCs) activate OXPHOS while undifferentiated human MSCs are more dependent on glycolysis^83^. Misra et al. show that BMSCs use OXPHOS for ATP generation in the early stage of differentiation, while mature osteoblasts depend on glycolysis^53^. Altering OXPHOS affects osteoblast differentiation and function. The deletion of Evolutionarily Conserved Signaling Intermediate in Toll pathways (ECSIT) in skeletal progenitors results in impaired OXPHOS, skeletal deformity, defects in the bone marrow niche and spontaneous fractures^84^, supporting the potential link between OXPHOS and osteoblast differentiation. Similarly, reducing OXPHOS by targeting the nuclear receptor PPARδ inhibits osteoblast differentiation in vitro and bone formation in vivo^85^. On the other hand, forced stimulation of OXPHOS leads to the activation and acetylation of β-catenin and promotes osteoblast differentiation^86^. Mitochondrial dysfunction impacts osteoblast formation and is linked with accelerated bone loss^87^. Lee et al. showed that mitochondrial malic enzyme sustains aerobic glycolysis, suggesting a functional coupling between the mitochondria and aerobic glycolysis in osteoblasts^60^. PTH treatment of primary calvarial cells or MC3T3E1 cells stimulates both aerobic glycolysis and oxidative phosphorylation^88^. A recent study shows that PTH regulates the function of the ETC through induction of mitochondrial complex I and II activity, while PTH acutely induces glycolysis in the presence of exogenous glucose^89^. When glycolysis is inhibited, osteoblasts increase OXPHOS to generate ATP, indicating the dynamic regulation between glycolysis and OXPHOS for the metabolic adaptation of osteoblasts.

### Bone cells and lipid metabolism

**Fig. 3:**
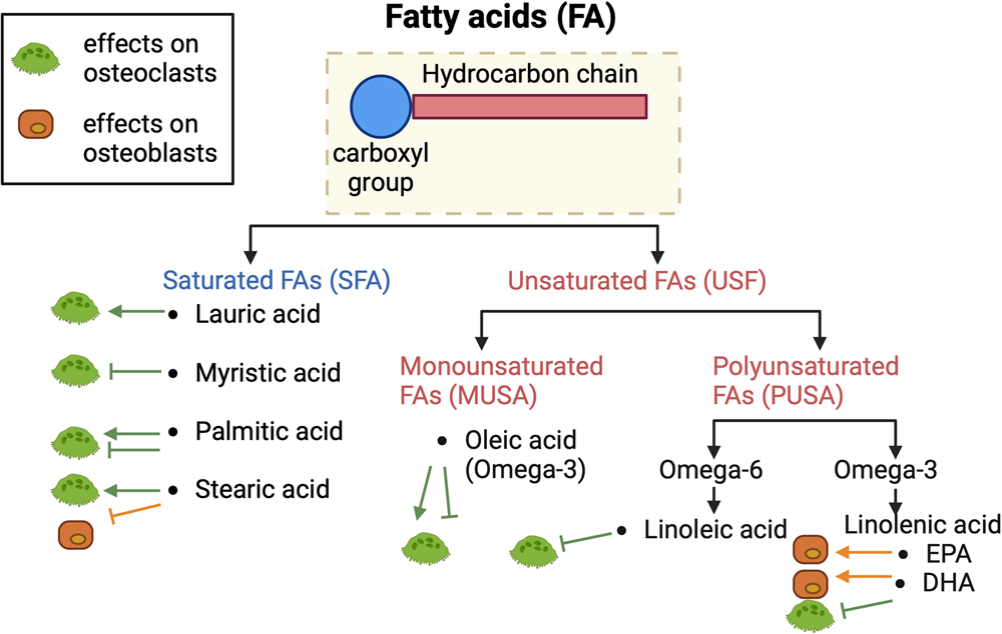
Fatty Acids regulate bone cells. Fatty acids are classified according to the presence and number of double bonds in their carbon chain: saturated fatty acids (SFA) contain no double bonds, monounsaturated fatty acids (MUFA) contain one, and polyunsaturated fatty acids (PUFA) contain more than one double bond. There are numerous types of SFA according to the length of their chain (containing 4–16 carbon atoms). PUFAs, such as alpha-linolenic acid (an omega-3 fatty acid) and linoleic acid (an omega-6 fatty acid), are called essential fatty acids because they are precursors to vitamins, cofactors, and derivatives, but our body cannot synthesize them. Fatty acids have both positive and negative effects on the regulation of osteoclasts (green cells and lines) and osteoblasts (orange cells and lines).

Lipids affect the function and differentiation of bone cells^90^. Lipid species such as cholesterols and fatty acids are circulating and are present in the bone marrow sera^91^. The oxidation of dietary lipids is a critical source of ATP for many cells and fuels bone remodeling. Cholesterols are delivered to the tissues by LDL through LDL receptor-mediated endocytosis^92^. The levels of LDL-cholesterol in the circulation have a strong inverse correlation with bone mineral density (BMD)^93^. Skeletal fatty acid utilization contributes to whole-body lipid homeostasis (Fig. 3). Dyslipidemia, a condition characterized by abnormal levels of lipids in the blood, can lead to an excessive accumulation of fat and adversely affect the skeletal system. Additionally, dyslipidemia indirectly affects bone loss by increasing parathyroid hormone, homocysteine, and lipid oxidation products, or by affecting estrogen, vitamin D, and K levels^94^. Among fatty acids, short-chain fatty acids (SCFAs) are produced in the gut by microbial fermentation of dietary fiber and provide a beneficial effect on bone homeostasis^95–97^. It is important to note that bone marrow is an important site for fat storage^98^, and marrow adipose tissues occupy over 70% of bone marrow space. Bone mass shows an inverse correlation with marrow fat^99^. Emerging evidence indicates the contribution of lipid species from membrane phospholipids, including sphingosine-1-phosphate, to skeletal health. Further studies are required to understand the local action of lipids in the bone environment and the underlying mechanism of lipid-mediated regulation of bone cells.

#### Osteoclasts and lipid metabolism

It is well-documented that lipids play a critical role in the survival, formation, and activity of osteoclasts^90^. Cholesterol can be obtained through both delivery and biosynthesis processes in osteoclasts. Increased cholesterol delivery via LDL significantly increases osteoclast viability^100^. Depleting cholesterol uptake in osteoclast via HDL or cyclodextrin treatment dose-dependently induced apoptosis^101^. The depletion of LDL suppresses osteoclast formation, and resupplying oxidized LDLs reverses the impaired osteoclastogenesis in the LDL-depleted serum^102^. Osteoclast lineage cells express genes involved in the cholesterol biosynthesis pathways, such as HMG-CoA reductase. Osteoclasts have lower expression of HMGCR compared to liver cells, and its expression level is not upregulated upon depletion of cholesterol from the plasma membrane^101^, suggesting the lack of feedback regulation of the cholesterol biosynthesis pathway during osteoclastogenesis. Statins are FDA-approved drugs targeting the cholesterol biosynthesis pathway and lowering cholesterols^103^. Clinical and animal studies show that statins increase BMD and prevent pathological bone loss and fractures^90,104^. These data suggest that both cholesterol uptake and de novo biosynthesis contribute to osteoclast differentiation, and reducing cholesterol levels may have a positive impact on bone health.

Different types of fatty acids (FA) have differential effects on osteoclast formation (Fig. 3). Common saturated fatty acids (SFAs), such as lauric acid and palmitic acid, are reported to enhance osteoclastic action by enhancing osteoclast survival^105^ or inducing osteoclast differentiation^106,107^. Palmitoleic acid inhibits RANKL-induced osteoclast differentiation from RAW264.7 through suppression of MAPK and NF-kB signaling^108^. Oleic acids, a mono-unsaturated FA, both positively and negatively regulate osteoclastogenesis^106,109^. Other unsaturated fatty acids (UFAs) inhibit human osteoclastogenesis by activating peroxisome proliferator-activated receptors (PPARs) through the inhibition of the RANKL signaling pathway^110^ or by reducing key signaling transduction pathways^111^. SCFAs are known to suppress osteoclastogenesis^95,96^. Although acetate does not protect mice from OVX-induced bone loss, feeding other SCFAs to ovariectomized mice, collagen-induced arthritic mice, or K/BxN serum transfer-induced arthritic mice protects mice from bone loss^95,112^. Medium-chain fatty acids such as capric acid also suppress osteoclastogenesis^113^. It is likely that different species of fatty acids have varying effects on osteoclastogenesis.

Recently, the importance of mitochondrial fatty acid β-oxidation (FAO) in osteoclast differentiation has been recognized. FAO converts long-chain FA to acetyl-CoA and plays a key role in the production of ATP and mitochondrial NADPH. Mature osteoclasts significantly increase FAO. Blocking FAO by deleting key enzymes of FAO, such as carnitine palmitoyltransferase 1a (Cpt1a) or Cpt2, in osteoclast progenitors suppresses in vitro osteoclastogenesis in both males and females. Interestingly, this negative impact of blocking FAO on in vivo osteoclastic bone resorption is observed only in female mice^109,114^, suggesting that FAO is differentially utilized by female-specific factors. Taken together, since diets and microbiomes have a significant contribution to the supply and processing of fatty acids, manipulating fatty acid metabolism can be a potential therapeutic strategy for bone diseases.

#### Osteoblasts and lipid metabolism

Osteoblast differentiation is primarily dependent on glycolysis (see “Bone Cells and Glycolysis” section). Emerging evidence shows that lipid metabolism is also important for osteoblast function, although its mechanism has not been clearly defined yet^115^. Cholesterol and its derivatives regulate osteoblast differentiation and their cellular functions^116,117^. However, the relationship between cholesterols and bone formation is quite complex. A series of studies have shown that exogenous cholesterol can inhibit in vitro osteoblast differentiation and the expression of osteoblastic genes^118^. In contrast, cholesterol treatment on mouse MSCs has been found to increase cell number and alkaline phosphatase (ALP) activity, along with an increase in gene expression of bone morphogenetic protein-1 (BMP1), Runx2, and Bglap2. Additionally, inhibiting acyl-CoA: cholesterol acyltransferase (ACAT) has been shown to attenuate osteogenesis, suggesting the essential role of cholesterol esters in osteoblast differentiation^116^. Oxidized LDLs inhibit osteoblast survival and mineralization^119^. Specific oxysterols, a product of cholesterol oxidation, show a pro-osteogenic effect on pluripotent mesenchymal cells^120^. Recent studies also show the dual role of cholesterol in bone formation. In ST2 bone marrow stromal cell lines, exogenous cholesterol treatment decreased the expression of osteoblast marker genes and ALP activity, while the physiological levels of endogenous cholesterol are critical for osteogenic response to Purmo^121^.

SFAs inhibit osteoblast differentiation and induce lipotoxicity and cell death. However, a recent study shows that endogenous FAs stored in lipid droplets can be utilized during osteoblast maturation^122^. Long-chain PUFAs such as EPA and DHA positively regulate osteoblast survival and differentiation. FAO increases in mature osteoblasts, and the Wnt-Lrp5 signaling pathway induces genes related to FAO. Mice lacking Lrp5 in osteoblasts and osteocytes exhibit a decrease in bone mass with increased body fat^123^. Cpt2 deletion with OCN-CRE impairs in vitro osteoblast differentiation and results in increased lactate production. However, FAO affects postnatal bone acquisition dominantly in female mice^124^. Further research is needed to discover factors that are specific to females and related to FAO. While the promoting effect of SCFAs on bone mass is evident, the direct role of SCFAs on bone formation is still controversial. Butyrate promotes bone formation. Acetate, one of the SCFAs, increases bone mass by enhancing bone formation in a T cell-dependent manner^97^. However, other studies show a minimal effect of SCFAs on osteoblasts. Taken together, lipid metabolism plays a key role in osteoblast function, although the source of fatty acids and the dual role of cholesterols need to be clarified. Furthermore, there is an increasing appreciation of the critical role of SCFAs in bone metabolism and inflammation, as well as the therapeutic potential of dietary fatty acids (FAs) in bone diseases.

## Nutrition and bone health

Nutrition plays a critical role in maintaining the health of our bones and metabolism in our bodies^125^. A well-balanced diet and sufficient intake of essential nutrients are crucial for optimal bone health. Adequate nutrition plays a significant role in postnatal bone formation, and metabolic disturbances can affect it.

The current research supports the idea that the consumption, digestion, and storage of fats play a crucial role in bone health. Obesity is characterized by an abnormal accumulation of excessive fat in the body, which is defined by a body mass index (BMI) over 30. A World Health Organization (WHO) report revealed that the prevalence of obesity in the European region has reached epidemic proportions with 59% of adults and nearly 1 in 3 children affected by overweight or obesity^126^. Obesity is a complex disorder associated with a wide range of complications and is a condition with a low-grade, systemic inflammatory state via the release of pro-inflammatory cytokines, such as leptin, TNF-alpha, and IL-6^127^. Obesity leads to other diseases, including diabetes, characterized by insulin insensitivity, cardiovascular diseases, and musculoskeletal disorders. There is an increased risk of fractures and bone fragility among obese individuals compared to normal-weight individuals^128^. However, the impact of obesity on bones is still controversial. Multiple population studies report a positive correlation between BMI and bone mass density (BMD) in both weight-bearing and non-weight-bearing bones for men and women^129–131^, suggesting that obesity is associated with increased bone mass in humans. The positive correlation between obesity and bone can be explained by multiple factors, such as an increase in load-bearing activity, increased estrogens released by adipose tissue, and/or an increase in mesenchymal progenitor cells that can differentiate into either osteoblasts or adipocytes^132^. In contrast, other studies report that obese subjects have a site-specific increase in BMD or have normal or slightly low BMD compared to non-obese subjects^133^. Romagnoli et al. show that the TBS (trabecular bone score) is inversely related to BMI^134^. A recent study demonstrates that obese postmenopausal women have higher bone resorption markers and lower bone formation markers than non-obese control subjects^135^, suggesting a reduction in trabecular bone and altered bone turnover markers may be related to an increased fracture risk in obese people. In addition, an inverse correlation between BMD and obesity has been documented in sarcopenic obesity and high-fat-fed animal mice^136,137^. Developing children and adults who are fed high-fat diets (aka Ketogenic diets) also observe a decrease in total BMD^138,139^. The mechanism underlying an increased risk of fracture in individuals with obesity and the interplay between metabolism-associated environment and bone cells are not fully characterized yet.

The uptake of key nutrients of bone, including vitamin D and calcium, affects bone mass^140^. Vitamin D is a steroid hormone that plays a vital role in maintaining calcium and phosphate homeostasis and calcium absorption. Vitamin D affects bone metabolism and metabolic diseases such as obesity, diabetes, and NAFLD. While vitamin D3 (cholecalciferol) is mainly produced from the skin by sunlight exposure, the synthesis of vitamin D3 varies by various factors such as age and season. Vitamin D also regulates non-skeletal tissues such as adipocytes, and adipocytes are a main vitamin D storage site. Individuals with obesity have lower levels of vitamin D in the circulation due to increased clearance of vitamin D^141^. Vitamin D deficiency leads to decreased calcium absorption, secondary hyperparathyroidism, high bone turnover, bone loss, bone fractures, and mineralization defects. Severe vitamin D deficiency causes osteomalacia in adults and rickets in children. However, the effect of vitamin D supplementation on BMD is still inconclusive and the optimal levels of vitamin D may differ by disease status or the levels of obesity of individuals. There are many other nutrient factors affecting bone and energy metabolism (reviewed in ref. ^142^), which are not discussed in this review.

## Prospects

Research over the past decades has established metabolism as a fundamental requirement underlying bone cell function in health and disease. However, we are just beginning to understand the effects of metabolic signaling on bone cell activation and how metabolic coordination works during bone cell differentiation. This review discusses the current understanding of the role of glycolysis, lipid metabolism, and OXPHOS in bone cell differentiation and function. However, other metabolic pathways, such as glutamine and amino acid metabolism (reviewed in ref. ^143^), also play an essential role in the differentiation and activity of bone cells.

Bone functions as an endocrine organ. Critical regulators of the skeleton, such as WNT, RANKL, osteoprotegerin (OPG), and sex hormones, are significantly affected by the nutrition and metabolic status of the body^144^. Moreover, the other direction from bone cells to the body has been revealed. Bone cells produce bioactive proteins which circulate through blood and target other organs, affecting their energy homeostasis^145^. One of the examples is osteocalcin^146^. Osteocalcin promotes glucose uptake, controls insulin sensitivity outside bone, and contributes to energy metabolism in the whole body. However, many questions still remain in the field. Our knowledge about the bioenergetics of osteocytes is currently very limited^147^. However, it is hard to isolate functional mitochondria or test metabolic analysis in osteocytes from mineralized tissue. Since these cells play a crucial role in the process of bone repair and regeneration, it is essential to understand how their metabolic changes affect their functions. The next question is how environmental cues associated with metabolism further shape bone remodeling. Extensive communication among bone cells plays a significant role in bone remodeling. However, we don’t fully understand how metabolism contributes to the complicate networks of bone cells. Moreover, multiple signaling pathways are activated or deactivated during the differentiation and function of bone cells. However, we still need to uncover how various metabolic pathways contribute to key signaling transduction pathways in bone cells. Energy metabolism generates metabolic intermediates used as substrates and co-factors for epigenetic enzymes, which shape the epigenetic landscape and regulate gene expression and cell fate decision^148^. However, the reciprocal regulation between cellular metabolism and gene expression in bone cells is still not extensively studied yet. Since transcriptomics and metabolomics of bone cells have already been studied, integrating the analysis of those pathways promises to reveal new biology and disease therapies. As many ways to target metabolic pathways have been developed, a better understanding of bone metabolism may lead to more personalized treatment protocols for bone diseases.

Taken together, the bioenergetic of bone cells plays a crucial role in maintaining healthy bones, promoting bone development and function, as well as the pathogenesis of bone diseases. The significance of bone in systemic bioenergetics highlights the need for future research on bone metabolism. This will help identify potential targets for pharmacological intervention, leading to improved management of not just bone diseases, but also systemic metabolic diseases.

## References

[1] Florencio-Silva, R., Sasso, G. R., Sasso-Cerri, E., Simoes, M. J. & Cerri, P. S. Biology of Bone Tissue: Structure, Function, and Factors That Influence Bone Cells. *Biomed. Res. Int.* **2015**, 421746 (2015). 26247020 10.1155/2015/421746 PMC4515490

[2] Karsenty, G. & Ferron, M. The contribution of bone to whole-organism physiology. *Nature* **481**, 314–320 (2012). 22258610 10.1038/nature10763 PMC9047059

[3] Peacock, M. Calcium metabolism in health and disease. *Clin. J. Am. Soc. Nephrol.* **5**, S23–S30 (2010). 20089499 10.2215/CJN.05910809

[4] Pinho, S. & Frenette, P. S. Haematopoietic stem cell activity and interactions with the niche. *Nat. Rev. Mol. Cell Biol.* **20**, 303–320 (2019). 30745579 10.1038/s41580-019-0103-9 PMC6483843

[5] Guntur, A. R. & Rosen, C. J. Bone as an endocrine organ. *Endocr. Pract.* **18**, 758–762 (2012). 22784851 10.4158/EP12141.RA PMC3571654

[6] DiGirolamo, D. J., Clemens, T. L. & Kousteni, S. The skeleton as an endocrine organ. *Nat. Rev. Rheumatol.* **8**, 674–683 (2012). 23045255 10.1038/nrrheum.2012.157

[7] Bolamperti, S., Villa, I. & Rubinacci, A. Bone remodeling: an operational process ensuring survival and bone mechanical competence. *Bone Res.* **10**, 48 (2022). 35851054 10.1038/s41413-022-00219-8 PMC9293977

[8] Park-Min, K. H. Mechanisms involved in normal and pathological osteoclastogenesis. *Cell Mol Life Sci.* **75**, 2519–2528 (2018). 29670999 10.1007/s00018-018-2817-9 PMC9809143

[9] Tsukasaki, M. & Takayanagi, H. Osteoimmunology: evolving concepts in bone-immune interactions in health and disease. *Nat. Rev. Immunol.* **19**, 626–642 (2019). 31186549 10.1038/s41577-019-0178-8

[10] Edwards, J. R. & Mundy, G. R. Advances in osteoclast biology: old findings and new insights from mouse models. *Nat. Rev. Rheumatol.* **7**, 235–243 (2011). 21386794 10.1038/nrrheum.2011.23

[11] Tsai, J., Kaneko, K., Suh, A. J., Bockman, R. & Park-Min, K. H. Origin of Osteoclasts: Osteoclast Precursor Cells. *J. Bone Metab.* **30**, 127–140 (2023). 37449346 10.11005/jbm.2023.30.2.127 PMC10346003

[12] McDonald, M. M. et al. Osteoclasts recycle via osteomorphs during RANKL-stimulated bone resorption. *Cell* **184**, 1330–1347.e1313 (2021). 33636130 10.1016/j.cell.2021.02.002 PMC7938889

[13] Park-Min, K. H. & Lorenzo, J. Osteoclasts: Other functions. *Bone* **165**, 116576 (2022). 36195243 10.1016/j.bone.2022.116576

[14] Mun, S. H., Park, P. S. U. & Park-Min, K. H. The M-CSF receptor in osteoclasts and beyond. *Exp. Mol. Med.* **52**, 1239–1254 (2020). 32801364 10.1038/s12276-020-0484-z PMC8080670

[15] Miller, P. D. Denosumab: anti-RANKL antibody. *Curr. Osteoporos. Rep.* **7**, 18–22 (2009). 19239825 10.1007/s11914-009-0004-5

[16] Kendler, D. L., Cosman, F., Stad, R. K. & Ferrari, S. Denosumab in the Treatment of Osteoporosis: 10 Years Later: A Narrative Review. *Adv. Ther.* **39**, 58–74 (2022). 34762286 10.1007/s12325-021-01936-y PMC8799550

[17] Park-Min, K. H. Metabolic reprogramming in osteoclasts. *Semin. Immunopathol.* **41**, 565–572 (2019). 31552471 10.1007/s00281-019-00757-0 PMC7671717

[18] Long, F. Building strong bones: molecular regulation of the osteoblast lineage. *Nat. Rev. Mol. Cell Biol.* **13**, 27–38 (2011). 22189423 10.1038/nrm3254

[19] Pittenger, M. F. et al. Multilineage potential of adult human mesenchymal stem cells. *Science* **284**, 143–147 (1999). 10102814 10.1126/science.284.5411.143

[20] Salhotra, A., Shah, H. N., Levi, B. & Longaker, M. T. Mechanisms of bone development and repair. *Nat. Rev. Mol. Cell Biol.* **21**, 696–711 (2020). 32901139 10.1038/s41580-020-00279-w PMC7699981

[21] Akiyama, H. et al. Osteo-chondroprogenitor cells are derived from Sox9 expressing precursors. *Proc. Natl Acad. Sci. USA* **102**, 14665–14670 (2005). 16203988 10.1073/pnas.0504750102 PMC1239942

[22] Komori, T. Regulation of Proliferation, Differentiation and Functions of Osteoblasts by Runx2. *Int. J. Mol. Sci.* **20**, 10.3390/ijms20071694 (2019). 10.3390/ijms20071694 PMC6480215 30987410

[23] Nakashima, K. et al. The novel zinc finger-containing transcription factor osterix is required for osteoblast differentiation and bone formation. *Cell* **108**, 17–29 (2002). 11792318 10.1016/s0092-8674(01)00622-5

[24] Wilson, C. Osteocytes, RANKL and bone loss. *Nat. Rev. Endocrinol.* **7**, 693 (2011). 21970841 10.1038/nrendo.2011.176

[25] Bellido, T. Osteocyte-driven bone remodeling. *Calcif. Tissue Int.* **94**, 25–34 (2014). 24002178 10.1007/s00223-013-9774-y PMC3947228

[26] Robling, A. G. & Bonewald, L. F. The Osteocyte: New Insights. *Annu. Rev. Physiol.* **82**, 485–506 (2020). 32040934 10.1146/annurev-physiol-021119-034332 PMC8274561

[27] Qin, L., Liu, W., Cao, H. & Xiao, G. Molecular mechanosensors in osteocytes. *Bone Res.* **8**, 23 (2020). 32550039 10.1038/s41413-020-0099-y PMC7280204

[28] Taubmann, J. et al. Metabolic reprogramming of osteoclasts represents a therapeutic target during the treatment of osteoporosis. *Sci. Rep.* **10**, 21020 (2020). 33273570 10.1038/s41598-020-77892-4 PMC7713370

[29] Indo, Y. et al. Metabolic regulation of osteoclast differentiation and function. *J. Bone Min. Res.* **28**, 2392–2399 (2013). 10.1002/jbmr.1976 23661628

[30] Karner, C. M. & Long, F. Glucose metabolism in bone. *Bone* **115**, 2–7 (2018). 28843700 10.1016/j.bone.2017.08.008 PMC6030501

[31] Ikeda, K. & Takeshita, S. The role of osteoclast differentiation and function in skeletal homeostasis. *J. Biochem.* **159**, 1–8 (2016). 26538571 10.1093/jb/mvv112 PMC4882648

[32] Lemma, S. et al. Energy metabolism in osteoclast formation and activity. *Int. J. Biochem. Cell Biol.* **79**, 168–180 (2016). 27590854 10.1016/j.biocel.2016.08.034

[33] Li, B. et al. Both aerobic glycolysis and mitochondrial respiration are required for osteoclast differentiation. *FASEB J.* **34**, 11058–11067 (2020). 32627870 10.1096/fj.202000771R

[34] Wilches-Buitrago, L., Viacava, P. R., Cunha, F. Q., Alves-Filho, J. C. & Fukada, S. Y. Fructose 1,6-bisphosphate inhibits osteoclastogenesis by attenuating RANKL-induced NF-κB/NFATc-1. *Inflammation Res.* **68**, 415–421 (2019). 10.1007/s00011-019-01228-w 30927049

[35] Ahn, H. et al. Accelerated Lactate Dehydrogenase Activity Potentiates Osteoclastogenesis via NFATc1 Signaling. *PLoS One* **11**, e0153886 (2016). 27077737 10.1371/journal.pone.0153886 PMC4831772

[36] Fan, J. et al. Quantitative flux analysis reveals folate-dependent NADPH production. *Nature* **510**, 298–302 (2014). 24805240 10.1038/nature13236 PMC4104482

[37] Zhang, L. et al. Arginine methylation of PPP1CA by CARM1 regulates glucose metabolism and affects osteogenic differentiation and osteoclastic differentiation. *Clin. Transl. Med.* **13**, e1369 (2023). 37649137 10.1002/ctm2.1369 PMC10468565

[38] Spencer, J. A. et al. Direct measurement of local oxygen concentration in the bone marrow of live animals. *Nature* **508**, 269–273 (2014). 24590072 10.1038/nature13034 PMC3984353

[39] Tan, J. K., Mohamad Hazir, N. S. & Alias, E. Impacts of Hypoxia on Osteoclast Formation and Activity: Systematic Review. *Int. J. Mol. Sci.* **22**, 10.3390/ijms221810146 (2021). 10.3390/ijms221810146 PMC8467526 34576310

[40] Tang, Y. et al. Mandibular osteotomy-induced hypoxia enhances osteoclast activation and acid secretion by increasing glycolysis. *J. Cell. Physiol.* **234**, 11165–11175 (2019). 30548595 10.1002/jcp.27765

[41] Morten, K. J., Badder, L. & Knowles, H. J. Differential regulation of HIF-mediated pathways increases mitochondrial metabolism and ATP production in hypoxic osteoclasts. *J. Pathol.* **229**, 755–764 (2013). 23303559 10.1002/path.4159 PMC3618370

[42] Meng, X., Wielockx, B., Rauner, M. & Bozec, A. Hypoxia-Inducible Factors Regulate Osteoclasts in Health and Disease. *Front. Cell Dev. Biol.* **9**, 658893 (2021). 33816509 10.3389/fcell.2021.658893 PMC8014084

[43] Hienz, S. A., Paliwal, S. & Ivanovski, S. Mechanisms of Bone Resorption in Periodontitis. *J. Immunol. Res.* **2015**, 615486 (2015). 26065002 10.1155/2015/615486 PMC4433701

[44] Murata, K. et al. Hypoxia-Sensitive COMMD1 Integrates Signaling and Cellular Metabolism in Human Macrophages and Suppresses Osteoclastogenesis. *Immunity* **47**, 66–79.e65 (2017). 28723554 10.1016/j.immuni.2017.06.018 PMC5568808

[45] Doi, K. et al. Role of Lysine-Specific Demethylase 1 in Metabolically Integrating Osteoclast Differentiation and Inflammatory Bone Resorption Through Hypoxia-Inducible Factor 1α and E2F1. *Arthritis Rheumatol.* **74**, 948–960 (2022). 35077015 10.1002/art.42074 PMC9156537

[46] Cohn, D. V. & Forscher, B. K. Aerobic metabolism of glucose by bone. *J. Biological Chem.* **237**, 615–618 (1962). 13880345

[47] Peck, W. A., Birge, S. J. Jr. & Fedak, S. A. Bone cells: biochemical and biological studies after enzymatic isolation. *Science* **146**, 1476–1477 (1964). 14208576 10.1126/science.146.3650.1476

[48] Borle, A. B., Nichols, N. & Nichols, G. Jr. Metabolic studies of bone in vitro. I. Normal bone. *J. Biological Chem.* **235**, 1206–1210 (1960). 13802861

[49] Wei, J. et al. Glucose Uptake and Runx2 Synergize to Orchestrate Osteoblast Differentiation and Bone Formation. *Cell* **161**, 1576–1591 (2015). 26091038 10.1016/j.cell.2015.05.029 PMC4475280

[50] Zoch, M. L., Abou, D. S., Clemens, T. L., Thorek, D. L. & Riddle, R. C. In vivo radiometric analysis of glucose uptake and distribution in mouse bone. *Bone Res.* **4**, 16004 (2016). 27088042 10.1038/boneres.2016.4 PMC4820746

[51] Guntur, A. R. et al. Osteoblast-like MC3T3-E1 Cells Prefer Glycolysis for ATP Production but Adipocyte-like 3T3-L1 Cells Prefer Oxidative Phosphorylation. *J. Bone Min. Res.* **33**, 1052–1065 (2018). 10.1002/jbmr.3390 PMC6002892 29342317

[52] Lee, S. Y., Abel, E. D. & Long, F. Glucose metabolism induced by Bmp signaling is essential for murine skeletal development. *Nat. Commun.* **9**, 4831 (2018). 30446646 10.1038/s41467-018-07316-5 PMC6240091

[53] Misra, B. B., Jayapalan, S., Richards, A. K., Helderman, R. C. M. & Rendina-Ruedy, E. Untargeted metabolomics in primary murine bone marrow stromal cells reveals distinct profile throughout osteoblast differentiation. *Metabolomics* **17**, 86 (2021). 34537901 10.1007/s11306-021-01829-9 PMC8450216

[54] Zoidis, E., Ghirlanda-Keller, C. & Schmid, C. Stimulation of glucose transport in osteoblastic cells by parathyroid hormone and insulin-like growth factor I. *Mol. Cell. Biochem.* **348**, 33–42 (2011). 21076856 10.1007/s11010-010-0634-z

[55] Li, Z. et al. Glucose Transporter-4 Facilitates Insulin-Stimulated Glucose Uptake in Osteoblasts. *Endocrinology* **157**, 4094–4103 (2016). 27689415 10.1210/en.2016-1583 PMC5086531

[56] Arponen, M., Jalava, N., Widjaja, N. & Ivaska, K. K. Glucose transporters GLUT1, GLUT3, and GLUT4 have different effects on osteoblast proliferation and metabolism. *Front. Physiol.* **13**, 1035516 (2022). 36523556 10.3389/fphys.2022.1035516 PMC9744933

[57] Chen, H. et al. Increased glycolysis mediates Wnt7b-induced bone formation. *FASEB J.* **33**, 7810–7821 (2019). 30913395 10.1096/fj.201900201RR PMC6593878

[58] Komarova, S. V., Ataullakhanov, F. I. & Globus, R. K. Bioenergetics and mitochondrial transmembrane potential during differentiation of cultured osteoblasts. *Am. J. Physiol. Cell Physiol.* **279**, C1220–1229 (2000). 11003602 10.1152/ajpcell.2000.279.4.C1220

[59] Guntur, A. R., Le, P. T., Farber, C. R. & Rosen, C. J. Bioenergetics during calvarial osteoblast differentiation reflect strain differences in bone mass. *Endocrinology* **155**, 1589–1595 (2014). 24437492 10.1210/en.2013-1974 PMC3990840

[60] Lee, W. C., Ji, X., Nissim, I. & Long, F. Malic Enzyme Couples Mitochondria with Aerobic Glycolysis in Osteoblasts. *Cell Rep.* **32**, 108108 (2020). 32905773 10.1016/j.celrep.2020.108108 PMC8183612

[61] Minami, E. et al. Lactate-induced histone lactylation by p300 promotes osteoblast differentiation. *PLoS One* **18**, e0293676 (2023). 38051708 10.1371/journal.pone.0293676 PMC10697613

[62] Nichols, F. C. & Neuman, W. F. Lactic acid production in mouse calvaria in vitro with and without parathyroid hormone stimulation: lack of acetazolamide effects. *Bone* **8**, 105–109 (1987). 3593606 10.1016/8756-3282(87)90078-0

[63] Rodan, G. A., Rodan, S. B. & Marks, S. C. Jr. Parathyroid hormone stimulation of adenylate cyclase activity and lactic acid accumulation in calvaria of osteopetrotic (ia) rats. *Endocrinology* **102**, 1501–1505 (1978). 217627 10.1210/endo-102-5-1501

[64] Neuman, W. F., Neuman, M. W. & Brommage, R. Aerobic glycolysis in bone: lactate production and gradients in calvaria. *Am. J. Physiol.* **234**, C41–C50 (1978). 623240 10.1152/ajpcell.1978.234.1.C41

[65] Felix, R., Neuman, W. F. & Fleisch, H. Aerobic glycolysis in bone: lactic acid production by rat calvaria cells in culture. *Am. J. Physiol.* **234**, C51–C55 (1978). 23680 10.1152/ajpcell.1978.234.1.C51

[66] Borle, A. B., Nichols, N. & Nichols, G. Jr. Metabolic studies of bone in vitro. II. The metabolic patterns of accretion and resorption. *J. Biological Chem.* **235**, 1211–1214 (1960). 13802862

[67] Esen, E., Lee, S. Y., Wice, B. M. & Long, F. PTH Promotes Bone Anabolism by Stimulating Aerobic Glycolysis via IGF Signaling. *J. Bone Min. Res.* **30**, 2137 (2015). 10.1002/jbmr.2556 PMC4825329 25990470

[68] Zhang, D. et al. The bone anabolic effects of irisin are through preferential stimulation of aerobic glycolysis. *Bone* **114**, 150–160 (2018). 29775761 10.1016/j.bone.2018.05.013

[69] Jin, Z. et al. Nitric oxide modulates bone anabolism through regulation of osteoblast glycolysis and differentiation. *J. Clin. Invest.* **131**, 10.1172/jci138935 (2021). 10.1172/JCI138935 PMC7919726 33373331

[70] Hong, M. et al. MiR-34a suppresses osteoblast differentiation through glycolysis inhibition by targeting lactate dehydrogenase-A (LDHA). *In vitro Cell. Dev. Biol. Animal* **56**, 480–487 (2020). 10.1007/s11626-020-00467-0 32719987

[71] Bae, S. et al. MYC-dependent oxidative metabolism regulates osteoclastogenesis via nuclear receptor ERRalpha. *J. Clin. Invest.* **127**, 2555–2568 (2017). 28530645 10.1172/JCI89935 PMC5490751

[72] Dudley, H. R. & Spiro, D. The Fine Structure of Bone Cells. *J. Biophys. Biochem. Cytol.* **11**, 627–649 (1961). 19866598 10.1083/jcb.11.3.627 PMC2225143

[73] Yang, M. & Vousden, K. H. Serine and one-carbon metabolism in cancer. *Nat. Rev. Cancer* **16**, 650–662 (2016). 27634448 10.1038/nrc.2016.81

[74] Ishii, K. A. et al. Coordination of PGC-1beta and iron uptake in mitochondrial biogenesis and osteoclast activation. *Nat. Med.* **15**, 259–266 (2009). 19252502 10.1038/nm.1910

[75] Shao, D. et al. PGC-1 beta-regulated mitochondrial biogenesis and function in myotubes is mediated by NRF-1 and ERR alpha. *Mitochondrion* **10**, 516–527 (2010). 20561910 10.1016/j.mito.2010.05.012

[76] Wei, W. et al. PGC1beta mediates PPARgamma activation of osteoclastogenesis and rosiglitazone-induced bone loss. *Cell Metab.* **11**, 503–516 (2010). 20519122 10.1016/j.cmet.2010.04.015 PMC3521515

[77] Zhang, Y. et al. PGC1beta Organizes the Osteoclast Cytoskeleton by Mitochondrial Biogenesis and Activation. *J. Bone Miner. Res.* **33**, 1114–1125 (2018). 29521005 10.1002/jbmr.3398 PMC6002881

[78] Sabini, E., Arboit, L., Khan, M. P., Lanzolla, G. & Schipani, E. Oxidative phosphorylation in bone cells. *Bone Rep.* **18**, 101688 (2023). 37275785 10.1016/j.bonr.2023.101688 PMC10238578

[79] Cao, S. et al. L-arginine metabolism inhibits arthritis and inflammatory bone loss. *Ann. Rheumatic Dis.* **83**, 72–87 (2024). 10.1136/ard-2022-223626 PMC10803985 37775153

[80] Passi-Even, L., Gazit, D. & Bab, I. Ontogenesis of ultrastructural features during osteogenic differentiation in diffusion chamber cultures of marrow cells. *J. Bone Min. Res.* **8**, 589–595 (1993). 10.1002/jbmr.5650080510 8511986

[81] Klein, B. Y., Gal, I., Hartshtark, Z. & Segal, D. Induction of osteoprogenitor cell differentiation in rat marrow stroma increases mitochondrial retention of rhodamine 123 in stromal cells. *J. Cell. Biochem.* **53**, 190–197 (1993). 8263035 10.1002/jcb.240530303

[82] Schilling, K., Brown, E. & Zhang, X. NAD(P)H autofluorescence lifetime imaging enables single cell analyses of cellular metabolism of osteoblasts in vitro and in vivo via two-photon microscopy. *Bone* **154**, 116257 (2022). 34781049 10.1016/j.bone.2021.116257 PMC8671374

[83] Shum, L. C., White, N. S., Mills, B. N., Bentley, K. L. & Eliseev, R. A. Energy Metabolism in Mesenchymal Stem Cells During Osteogenic Differentiation. *Stem Cells Dev.* **25**, 114–122 (2016). 26487485 10.1089/scd.2015.0193 PMC4733323

[84] Lin, C. et al. Impaired mitochondrial oxidative metabolism in skeletal progenitor cells leads to musculoskeletal disintegration. *Nat. Commun.* **13**, 6869 (2022). 36369293 10.1038/s41467-022-34694-8 PMC9652319

[85] Muller, D. I. H. et al. PPARdelta-mediated mitochondrial rewiring of osteoblasts determines bone mass. *Sci. Rep.* **10**, 8428 (2020). 32439961 10.1038/s41598-020-65305-5 PMC7242479

[86] Shares, B. H., Busch, M., White, N., Shum, L. & Eliseev, R. A. Active mitochondria support osteogenic differentiation by stimulating beta-catenin acetylation. *J. Biol. Chem.* **293**, 16019–16027 (2018). 30150300 10.1074/jbc.RA118.004102 PMC6187642

[87] Dobson, P. F. et al. Mitochondrial dysfunction impairs osteogenesis, increases osteoclast activity, and accelerates age related bone loss. *Sci. Rep.* **10**, 11643 (2020). 32669663 10.1038/s41598-020-68566-2 PMC7363892

[88] Esen, E., Lee, S. Y., Wice, B. M. & Long, F. PTH Promotes Bone Anabolism by Stimulating Aerobic Glycolysis via IGF Signaling. *J. Bone Miner. Res.* **30**, 1959–1968 (2015). 25990470 10.1002/jbmr.2556 PMC4825329

[89] DeMambro, V. E., Tian, L., Karthik, V., Rosen, C. J. & Guntur, A. R. Effects of PTH on osteoblast bioenergetics in response to glucose. *Bone Rep.* **19**, 101705 (2023). 37576927 10.1016/j.bonr.2023.101705 PMC10412867

[90] Kim, H., Oh, B. & Park-Min, K. H. Regulation of Osteoclast Differentiation and Activity by Lipid Metabolism. *Cells* **10**, 10.3390/cells10010089 (2021). 10.3390/cells10010089 PMC7825801 33430327

[91] Pino, A. M., Miranda, M., Figueroa, C., Rodriguez, J. P. & Rosen, C. J. Qualitative Aspects of Bone Marrow Adiposity in Osteoporosis. *Front. Endocrinol.* **7**, 139 (2016). 10.3389/fendo.2016.00139 PMC5078474 27826285

[92] Brown, A. L. Jr., Taylor, W. F. & Carter, R. E. The reliability of measures of amphibole fiber concentration in water. *Environ. Res.* **12**, 150–160 (1976). 964214 10.1016/0013-9351(76)90018-9

[93] Yu, H. M., Liu, B., Costantini, F. & Hsu, W. Impaired neural development caused by inducible expression of Axin in transgenic mice. *Mech. Dev.* **124**, 146–156 (2007). 17123792 10.1016/j.mod.2006.10.002 PMC1847614

[94] Wang, B., Wang, H., Li, Y. & Song, L. Lipid metabolism within the bone micro-environment is closely associated with bone metabolism in physiological and pathophysiological stages. *Lipids Health Dis.* **21**, 5 (2022). 34996476 10.1186/s12944-021-01615-5 PMC8742318

[95] Lucas, S. et al. Short-chain fatty acids regulate systemic bone mass and protect from pathological bone loss. *Nat. Commun.* **9**, 55 (2018). 29302038 10.1038/s41467-017-02490-4 PMC5754356

[96] Rahman, M. M. et al. Two histone deacetylase inhibitors, trichostatin A and sodium butyrate, suppress differentiation into osteoclasts but not into macrophages. *Blood* **101**, 3451–3459 (2003). 12511413 10.1182/blood-2002-08-2622

[97] Tyagi, A. M. et al. The Microbial Metabolite Butyrate Stimulates Bone Formation via T Regulatory Cell-Mediated Regulation of WNT10B Expression. *Immunity* **49**, 1116–1131.e1117 (2018). 30446387 10.1016/j.immuni.2018.10.013 PMC6345170

[98] Cawthorn, W. P. & Scheller, E. L. Editorial: Bone Marrow Adipose Tissue: Formation, Function, and Impact on Health and Disease. *Front. Endocrinol.* **8**, 112 (2017). 10.3389/fendo.2017.00112 PMC5447009 28611729

[99] Justesen, J. et al. Adipocyte tissue volume in bone marrow is increased with aging and in patients with osteoporosis. *Biogerontology* **2**, 165–171 (2001). 11708718 10.1023/a:1011513223894

[100] Sato, T., Morita, I. & Murota, S. Involvement of cholesterol in osteoclast-like cell formation via cellular fusion. *Bone* **23**, 135–140 (1998). 9701472 10.1016/s8756-3282(98)00082-9

[101] Luegmayr, E. et al. Osteoclast formation, survival and morphology are highly dependent on exogenous cholesterol/lipoproteins. *Cell Death Differ.* **11**, S108–S118 (2004). 15017384 10.1038/sj.cdd.4401399

[102] Okayasu, M. et al. Low-density lipoprotein receptor deficiency causes impaired osteoclastogenesis and increased bone mass in mice because of defect in osteoclastic cell-cell fusion. *J. Biol. Chem.* **287**, 19229–19241 (2012). 22500026 10.1074/jbc.M111.323600 PMC3365955

[103] Pedersen, T. R. & Tobert, J. A. Simvastatin: a review. *Expert Opin. Pharmacother.* **5**, 2583–2596 (2004). 15571475 10.1517/14656566.5.12.2583

[104] Wang, Z., Li, Y., Zhou, F., Piao, Z. & Hao, J. Effects of Statins on Bone Mineral Density and Fracture Risk: A PRISMA-compliant Systematic Review and Meta-Analysis. *Medicine* **95**, e3042 (2016). 27258488 10.1097/MD.0000000000003042 PMC4900696

[105] Oh, S. R. et al. Saturated fatty acids enhance osteoclast survival. *J. Lipid Res.* **51**, 892–899 (2010). 20388920 10.1194/jlr.M800626 PMC2853456

[106] Drosatos-Tampakaki, Z. et al. Palmitic acid and DGAT1 deficiency enhance osteoclastogenesis, while oleic acid-induced triglyceride formation prevents it. *J. Bone Min. Res.* **29**, 1183–1195 (2014). 10.1002/jbmr.2150 PMC4945760 24272998

[107] Cornish, J. et al. Modulation of osteoclastogenesis by fatty acids. *Endocrinology* **149**, 5688–5695 (2008). 18617622 10.1210/en.2008-0111

[108] van Heerden, B., Kasonga, A., Kruger, M. C. & Coetzee, M. Palmitoleic Acid Inhibits RANKL-Induced Osteoclastogenesis and Bone Resorption by Suppressing NF-kappaB and MAPK Signalling Pathways. *Nutrients* **9**, 10.3390/nu9050441 (2017). 10.3390/nu9050441 PMC5452171 28452958

[109] Song, C. et al. Sexual dimorphism of osteoclast reliance on mitochondrial oxidation of energy substrates in the mouse. *JCI Insight* **8**, 10.1172/jci.insight.174293 (2023). 10.1172/jci.insight.174293 PMC10807709 37917194

[110] Kasonga, A., Kruger, M. C. & Coetzee, M. Activation of PPARs Modulates Signalling Pathways and Expression of Regulatory Genes in Osteoclasts Derived from Human CD14+ Monocytes. *Int. J. Mol. Sci.* **20**, 10.3390/ijms20071798 (2019). 10.3390/ijms20071798 PMC6479901 30979019

[111] Kim, H. J. et al. Docosahexaenoic acid signaling attenuates the proliferation and differentiation of bone marrow-derived osteoclast precursors and promotes apoptosis in mature osteoclasts. *Cell. Signal.* **29**, 226–232 (2017). 27836739 10.1016/j.cellsig.2016.11.007

[112] Yang, K. L. et al. Short Chain Fatty Acids Mitigate Osteoclast-Mediated Arthritic Bone Remodelling. *Arthritis Rheumatol.*, 10.1002/art.42765 (2023). 10.1002/art.42765 PMC10965381 37994265

[113] Kwon, J. O., Jin, W. J., Kim, B., Kim, H. H. & Lee, Z. H. Myristoleic acid inhibits osteoclast formation and bone resorption by suppressing the RANKL activation of Src and Pyk2. *Eur. J. Pharmacol.* **768**, 189–198 (2015). 26528796 10.1016/j.ejphar.2015.10.053

[114] Kushwaha, P. et al. Mitochondrial fatty acid beta-oxidation is important for normal osteoclast formation in growing female mice. *Front. Physiol.* **13**, 997358 (2022). 36187756 10.3389/fphys.2022.997358 PMC9515402

[115] Lee, W. C., Guntur, A. R., Long, F. & Rosen, C. J. Energy Metabolism of the Osteoblast: Implications for Osteoporosis. *Endocr. Rev.* **38**, 255–266 (2017). 28472361 10.1210/er.2017-00064 PMC5460680

[116] Li, H., Guo, H. & Li, H. Cholesterol loading affects osteoblastic differentiation in mouse mesenchymal stem cells. *Steroids* **78**, 426–433 (2013). 23395977 10.1016/j.steroids.2013.01.007

[117] Parhami, F. et al. Role of the cholesterol biosynthetic pathway in osteoblastic differentiation of marrow stromal cells. *J. Bone Min. Res.* **17**, 1997–2003 (2002). 10.1359/jbmr.2002.17.11.1997 12412807

[118] Yin, W., Li, Z. & Zhang, W. Modulation of Bone and Marrow Niche by Cholesterol. *Nutrients* **11**, 10.3390/nu11061394 (2019). 10.3390/nu11061394 PMC6628005 31234305

[119] Maziere, C. et al. Oxidized low density lipoprotein inhibits phosphate signaling and phosphate-induced mineralization in osteoblasts. Involvement of oxidative stress. *Biochim. Biophys. Acta* **1802**, 1013–1019 (2010). 20667472 10.1016/j.bbadis.2010.07.010

[120] Kha, H. T. et al. Oxysterols regulate differentiation of mesenchymal stem cells: pro-bone and anti-fat. *J. Bone Miner. Res.* **19**, 830–840 (2004). 15068507 10.1359/JBMR.040115

[121] Li, K. et al. A dual role of cholesterol in osteogenic differentiation of bone marrow stromal cells. *J. Cell. Physiol.* **234**, 2058–2066 (2019). 30317648 10.1002/jcp.27635

[122] Nandy, A. et al. Lipolysis supports bone formation by providing osteoblasts with endogenous fatty acid substrates to maintain bioenergetic status. *Bone Res.* **11**, 62 (2023). 38001111 10.1038/s41413-023-00297-2 PMC10673934

[123] Frey, J. L. et al. Wnt-Lrp5 signaling regulates fatty acid metabolism in the osteoblast. *Mol. Cell Biol.* **35**, 1979–1991 (2015). 25802278 10.1128/MCB.01343-14 PMC4420919

[124] Kim, S. P. et al. Fatty acid oxidation by the osteoblast is required for normal bone acquisition in a sex- and diet-dependent manner. *JCI Insight* **2**, 10.1172/jci.insight.92704 (2017). 10.1172/jci.insight.92704 PMC5621897 28814665

[125] Jensen, V. F. H., Molck, A. M., Dalgaard, M., McGuigan, F. E. & Akesson, K. E. Changes in bone mass associated with obesity and weight loss in humans: Applicability of animal models. *Bone* **145**, 115781 (2021). 33285255 10.1016/j.bone.2020.115781

[126] Kelly, T., Yang, W., Chen, C. S., Reynolds, K. & He, J. Global burden of obesity in 2005 and projections to 2030. *Int. J. Obes.* **32**, 1431–1437 (2008). 10.1038/ijo.2008.102 18607383

[127] Makki, K., Froguel, P. & Wolowczuk, I. Adipose tissue in obesity-related inflammation and insulin resistance: cells, cytokines, and chemokines. *ISRN Inflamm.* **2013**, 139239 (2013). 24455420 10.1155/2013/139239 PMC3881510

[128] Compston, J. E. et al. Obesity is not protective against fracture in postmenopausal women: GLOW. *Am. J. Med.* **124**, 1043–1050 (2011). 22017783 10.1016/j.amjmed.2011.06.013 PMC4897773

[129] Sukumar, D. et al. Obesity alters cortical and trabecular bone density and geometry in women. *Osteoporos. Int.* **22**, 635–645 (2011). 20533027 10.1007/s00198-010-1305-3 PMC2994953

[130] Felson, D. T., Zhang, Y., Hannan, M. T. & Anderson, J. J. Effects of weight and body mass index on bone mineral density in men and women: the Framingham study. *J. Bone Miner. Res.* **8**, 567–573 (1993). 8511983 10.1002/jbmr.5650080507

[131] Cherif, R. et al. Positive Association of Obesity and Insulin Resistance With Bone Mineral Density in Tunisian Postmenopausal Women. *J. Clin. Densitom.* **21**, 163–171 (2018). 28687244 10.1016/j.jocd.2017.05.015

[132] Hu, L. et al. Mesenchymal Stem Cells: Cell Fate Decision to Osteoblast or Adipocyte and Application in Osteoporosis Treatment. *Int. J. Mol. Sci*. **19**, 10.3390/ijms19020360 (2018). 10.3390/ijms19020360 PMC5855582 29370110

[133] Rinonapoli, G. et al. Obesity and Bone: A Complex Relationship. *Int. J. Mol. Sci.* **22**, 10.3390/ijms222413662 (2021). 10.3390/ijms222413662 PMC8706946 34948466

[134] Romagnoli, E. et al. Assessment of trabecular bone score (TBS) in overweight/obese men: effect of metabolic and anthropometric factors. *Endocrine* **54**, 342–347 (2016). 26815904 10.1007/s12020-016-0857-1

[135] Lopez-Gomez, J. J. et al. Influence of Obesity on Bone Turnover Markers and Fracture Risk in Postmenopausal Women. *Nutrients* **14**, 10.3390/nu14081617 (2022). 10.3390/nu14081617 PMC9029584 35458178

[136] Ali, D. et al. High-fat diet-induced obesity augments the deleterious effects of estrogen deficiency on bone: Evidence from ovariectomized mice. *Aging Cell* **21**, e13726 (2022). 36217558 10.1111/acel.13726 PMC9741509

[137] Silva, M. J. et al. Effects of High-Fat Diet and Body Mass on Bone Morphology and Mechanical Properties in 1100 Advanced Intercross Mice. *J. Bone Miner. Res.* **34**, 711–725 (2019). 30615803 10.1002/jbmr.3648 PMC6879418

[138] Corwin, R. L., Hartman, T. J., Maczuga, S. A. & Graubard, B. I. Dietary saturated fat intake is inversely associated with bone density in humans: analysis of NHANES III. *J. Nutr.* **136**, 159–165 (2006). 16365076 10.1093/jn/136.1.159

[139] Simm, P. J. et al. The effect of the ketogenic diet on the developing skeleton. *Epilepsy Res.* **136**, 62–66 (2017). 28778055 10.1016/j.eplepsyres.2017.07.014

[140] Polzonetti, V., Pucciarelli, S., Vincenzetti, S. & Polidori, P. Dietary Intake of Vitamin D from Dairy Products Reduces the Risk of Osteoporosis. *Nutrients* **12**, 10.3390/nu12061743 (2020). 10.3390/nu12061743 PMC7353177 32532150

[141] Bachmann, K. N. Responses to Vitamin D Supplementation in Individuals With Overweight and Obesity. *JAMA Netw. Open* **6**, e2250695 (2023). 36648948 10.1001/jamanetworkopen.2022.50695

[142] Palacios, C. The role of nutrients in bone health, from A to Z. *Crit. Rev. Food Sci. Nutr.* **46**, 621–628 (2006). 17092827 10.1080/10408390500466174

[143] Devignes, C. S., Carmeliet, G. & Stegen, S. Amino acid metabolism in skeletal cells. *Bone Rep.* **17**, 101620 (2022). 36120644 10.1016/j.bonr.2022.101620 PMC9475269

[144] Karsenty, G. Convergence between bone and energy homeostases: leptin regulation of bone mass. *Cell Metab.* **4**, 341–348 (2006). 17084709 10.1016/j.cmet.2006.10.008

[145] Zhou, R. et al. Endocrine role of bone in the regulation of energy metabolism. *Bone Res.* **9**, 25 (2021). 34016950 10.1038/s41413-021-00142-4 PMC8137703

[146] Moser, S. C. & van der Eerden, B. C. J. Osteocalcin-A Versatile Bone-Derived Hormone. *Front. Endocrinol.* **9**, 794 (2018). 10.3389/fendo.2018.00794 PMC6335246 30687236

[147] Villasenor, A. et al. Metabolomics reveals citric acid secretion in mechanically-stimulated osteocytes is inhibited by high glucose. *Sci. Rep.* **9**, 2295 (2019). 30783155 10.1038/s41598-018-38154-6 PMC6381120

[148] Chen, C., Wang, Z. & Qin, Y. Connections between metabolism and epigenetics: mechanisms and novel anti-cancer strategy. *Front. Pharmacol.* **13**, 935536 (2022). 35935878 10.3389/fphar.2022.935536 PMC9354823

